# Peroxisome Proliferators Activated Receptor (PPAR) agonists activate hepatitis B virus replication in vivo

**DOI:** 10.1186/s12985-017-0765-x

**Published:** 2017-05-25

**Authors:** Lingyao Du, Yuanji Ma, Miao Liu, Libo Yan, Hong Tang

**Affiliations:** Center of Infectious Diseases, West China Hospital of Sichuan University, Chengdu, 610041 China

**Keywords:** PPAR, Agonist, HBV, Replication

## Abstract

**Background:**

PPAR agonists are often used in HBV infected patients with metabolic disorders. However, as liver-enriched transcriptional factors, PPARs would activate HBV replication. Risks exsit in such patients. This study aimed to assess the influence of commonly used synthetic PPAR agonists on hepatitis B virus (HBV) transcription, replication and expression through HBV replicative mouse models, providing information for physicians to make necessary monitoring and therapeutic adjustment when HBV infected patients receive PPAR agonists treatment.

**Methods:**

The HBV replicative mouse model was established by hydrodynamic injection of HBV replicative plasmid and the mice were divided into four groups and treated daily for 3 days with saline, PPAR pan-agonist (bezafibrate), PPARα agonist (fenofibrate) and PPARγ agonist (rosiglitazone) respectively. Their serum samples were collected for ECLIA analysis of HBsAg and HBeAg and real-time PCR analysis of Serum HBV DNA. The liver samples were collected for DNA (Southern) filter hybridization of HBV replication intermediates, real-time PCR analysis of HBV mRNA and immunohistochemistry (IHC) analysis of hepatic HBcAg. The alternation of viral transcription, replication and expression were compared in these groups.

**Result:**

Serum HBsAg, HBeAg and HBV DNA were significantly elevated after PPAR agonist treatment. So did the viral replication intermediates in mouse livers. HBV mRNA was also significantly increased by these PPAR agonists, implying that PPAR agonists activate HBV replication at transcription level. Moreover, hepatic HBcAg expression in mouse livers with PPAR agonist treatment was elevated as well.

**Conclusion:**

Our in vivo study proved that synthetic PPAR agonists bezafibrate, fenofibrate and rosiglitazone would increase HBV replication. It suggested that when HBV infected patients were treated with PPARs agonists because of metabolic diseases, HBV viral load should be monitored and regimens may need to be adjusted, an antiviral therapy may be added.

**Electronic supplementary material:**

The online version of this article (doi:10.1186/s12985-017-0765-x) contains supplementary material, which is available to authorized users.

## Background

Peroxisome proliferator-activated receptors (PPARs) are cytokines in inflammatory and metabolic pathways. They work not only in response to nutritional input but also to inflammatory stimulation. Three isolates, including PPARα, PPAR β/δ, and PPARγ, were identified by now [1]. Although in the same family, each PPAR has its own unique characteristics. PPARα mainly locates in liver and it regulates fatty acid transport, β-oxidation and ketogenesis. PPAR β/δ aggregates in skeletal muscles, and it regulates fatty acid catabolism and glycolytic-to-oxidative switchover. And PPARγ is highly expressed in adipose tissues and involved in fat storage and adipokine secretion. Because of their critical roles in inflammatory and metabolic pathways, synthetic PPAR agonists are widely used in patients with obesity associated disorders including dyslipidemia, type 2 diabetes mellitus (T2DM) and so on. For example, patients with hypertriglyceridemia commonly receive treatment of fibrates, a kind of PPARα agonists, and patients with T2DM often receive treatment of thiazolidinediones, a kind of PPARγ agonists that worked as insulin-sensitizers. Though PPAR β/δ agonists are not clinically used nowadays, bezafibrate, is a confirmed pan-agonist in use that activated PPARα, PPAR β/δ and PPARγ [2, 3].

As hepatitis B virus is globally epidemic, some patients with metabolic disorders also have HBV infection. And some studies have suggested that dyslipidemia and diabetes may increase risk of advanced liver diseases in HBV infected patients [4–6]. Therefore, PPAR agonists are often used in these patients. However, PPARs are the liver-enriched transcriptional factors, and they belong to nuclear hormone receptor superfamily. Tang’s group found that transfection of PPARα expression plasmid into non-liver cells endowed the cells with capacity to support HBV replication [7]. It noticed that PPARs could be related to HBV replication. Soon after Tang, studies confirmed that the activation of PPARα enhance viral replication in hepatocellular cells and transgenic mouse models [8, 9]. And activation of PPARγ was also proved to enhance viral replication in HBV stably transfected cells [10].

These studies strongly suggested that clinically used PPAR agonists may rise the risk of activating viral replication in HBV infected patients with metabolic disorders. But direct observations on the effects from these drugs to viral replication remain insufficient. And there are conflicting findings too: The Wakui study suggested that PPARγ could inhibit HBV replication in vitro through another more effective pathway [11]. Based on these situations, an assessment in vivo would bring us a clue.

Therefore, the aim of this study was to assess in vivo the influence of commonly used PPAR agonists on HBV transcription, replication and expression. It could provide more information on better management of HBV infected patients with metabolic disorders, especially management on the adjustment of regimens and monitoring of the patients.

## Methods

### Mouse models and treatments

Hydrodynamic HBV replicative mouse model was applied in this study. It was established with HBV replicative plasmid pHBV4.1 and SPF grade male BALB/c mice with age of 7–9 weeks and weight of 18–20 g. HBV replicative plasmid is applicable to study the viral biological characteristics and the influence from chemical agents to virus. Such plasmid can replicate effectively both in vitro and in vivo. It contains 1.3 copies of HBV genome with 4.1Kb in length in its multiple cloning sites: besides 3.2Kb of the HBV genome, a 0.9Kb of the repeating sequence from upstream of Enhance I and X promoter to downstream Poly A is integrated. And the plasmid usually named pHBV4.1. A solution of 10 μg naked replicative plasmid with a volume over total blood volume of a mouse (about 8% of weight) was injected into BALB/C mouse via the tail vein within 5 to 8 s [12]. Thus sufficient plasmid would enter hepatic sinusoid and be engulfed by hepatocytes. Such mouse model provided transient viral RNA transcription, DNA replication, and protein expression lasting for 10 days with a peak around day 3. It fulfilled the requirement of studies in the drug induced regulation on viral replication and expression [13].

Three clinically used PPAR agonists including two fibrates and one thiazolidinedione targeting on three kinds of PPARs were selected in this study. They are bezafibrate (TASLY PHARMACEUTICAL, Tianjin) of pan PPARs agonists, fenofibrate (RECIPHARM FONTAINE, France) of PPARα agonists and rosiglitazone (GlaxoSmithKline, US) of PPARγ agonists. Saline were selected as control here. Because when we analyzed the influence from these PPAR agonists to HBV replication, a baseline HBV replication level in HBV replicative mouse model without drug induced regulation was needed. A hydrodynamically injected mouse with saline treatment could providing such a reference level of HBV replication while balancing the influence from experimental operation procedures.

Established HBV replicative mouse models were divided into four groups with three mice in each group. Then they are treated with these three drugs and saline through gavage respectively each day. The equivalent dose of drugs in a mouse was calculated through the formula “dose in mouse ≈ dose in patients / 60 kg × 9.01”[13, 14]. The detailed information of drug doses administrated in each groups were shown in Table 1. Clinical tablets of bezafibrate, fenofibrate and rosiglitazone were grinded and dissolved in saline. The final concentration of the three PPAR agonist solutions were: 1.2 mg/mL, 1.2 mg/mL and 0.024 mg/mL respectively. According to the dosage calculating formula acquire from Handbook of Laboratory Animal Science, the daily gavage volume was 500 μL for each mouse. The administration of drugs started 12 h after hydrodynamic injection of HBV replicative plasmid. The gavage was proceeded once daily and lasted for 3 days. Six hours after the third gavage, their sera and livers were collected for sequential detections.

**Table 1:** The doses of drugs in each groups

Groups	Drug doses
A: Saline	-
B: Bezafibrate	30 mg/kg.d
C: Fenofibrate	30 mg/kg.d
D: Rosiglitazone	0.6 mg/kg.d

### Serum HBV antigen analysis

Cobas HBsAg II quant detection kit and Cobas HBeAg detection kit (Roche Diagnostics GmbH) were used to quantify the serum HBsAg and HBeAg expression in mouse models through electrochemiluminescence immunoassay (ECLIA). In each mouse, 50 μL serum was used for HBsAg detection and 35 μL serum was used for HBeAg detection. The operation was implemented in chemiluminescence immunoassay analyzer Cobas e 601 (Roche Diagnostics GmbH) according to manufacturer’s instructions.

### Serum HBV DNA analysis

In each mouse, 100 μL serum was applied to extract HBV DNA with Viral DNA Extraction Kit (BioTeke, China) according to instructions. And the products were quantified by real-time PCR with Diagnostic Kit for Quantification of Hepatitis B Virus DNA (PCR-Fluorescence Probing) (Shanghai Kehua Bio., China) in Roche LightCycler96 (Roche diagnostic, GmbH). The reaction volume for each sample was 30 μL consisting of 18 μL pre-mixed reaction buffer and 12 μL extraction product. The mixture was pre-denatured at 94°C for 120 s, followed by 40 cycles of 94°C for 10 s and 60°C for 30 s, then cooling at 35°C for 10s.

### HBV replication intermediates analysis

HBV replication intermediates in the mouse livers were isolated through previously described method [12, 13]. One hundred and twenty micrograms of liver tissue in each sample was lysed for isolation and the isolated DNA replication intermediates were dissolved in 30 μL 10 mmol/L Tris hydrochloride (pH 8.0) and 1 mmol/L EDTA. DNA (Southern) filter hybridization was performed with the 30 μL viral replication intermediates. Filter was probed with DIG Luminescent Detection Kit (Roche Applied Science) labeled full-length HBV genomic DNA (serotype ayw) and the detected replication intermediates were qualified in image analysis system (Quantity One, LifeScience, USA).

### HBV mRNA analysis

Fifty micrograms of liver tissue in each sample was lysed in TRIzol reagents (Life Technologies, USA). And total RNA were extracted according to the reagents’ instructions. With RNA reverse transcription kit (PrimeScript™ RT reagent Kit with gDNA Eraser, TAKARA BIO INC. Dalian), total RNA were reversely transcribed into cDNA. Then a pair of primers locating X gene of HBV genome (HBV-X-F CCTTCTTACTCTACCGTTCC, HBV-X-R GACCAATTTATGCCTACAGCC) were used to analyze HBV mRNA [15]. Another pair of primers locating GAPDH gene (MGAPDH-F GAGTGTTTCCTCGTCCCGTA, MGAPDH-R GAGGTCAATGAAGGGGTCGTT) were used to amplify the internal reference. The HBV mRNA level were detected through real-time PCR with Fast Start Universal SYBR Green Master in LightCycler96 (Roche diagnostic, GmbH). The amplification started with pre-incubation at 95°C for 600 s, followed by 40 cycles of 95°C for 10 s, 60 for 10 s and 72°C for 40 s. After melting and cooling, the Cq values were acquired to calculate the relative HBV transcription level in mouse liver tissues.

### Hepatic HBV antigen analysis

HBcAg in the liver was stained through IHC. Rabbit anti-HBc (NEOMARKERS) primary antibody and Polymer-HRP Anti-Rabbit (Zhongshan, Beijing) secondary antibody were applied. After stained with 3', 3'-diaminobenzidine tetrahydrochloride (DAB) and conterstained with hematoxylin, the sections were mounted and evaluated. Positive stain of HBcAg presented as particles in the hepatocytes. The percentage of positive hepatocytes and their staining intensity were evaluated. Axiotis scoring criteria was applied here. Percentage score ranged from 0 to 4, representing 0–10%, 11–25%, 26–50%, 51–75% and 76–100% respectively. Intensity score ranged from 0 to 3, representing no color, yellow, brown and tan. The sum of the percentage score and the intensitiy score equaled the sum score. And five different sum scores from random high power field (x400) were acquired for a mean sum score. The assessment was implemented by two pathologists unaware of the tissue section arrangement. If there is a difference in their opinions, extra mean would be calculated with the two mean sum for final score.

### Statistical analysis

All the data in three independent experiments were collected and analyzed in SPSS 18.00. All the measurement data went through normality test first. If the data were normal, they﻿ would be expressed with mean ± standard deviation (SD), analyzed with t test. If they were﻿﻿ non-normal, they﻿ would be expressed with median ± interquartile range (IQR), analyzed with u test. A comparison between normal data and non-normal data was also conducted via u test. A *p* value < 0.05 was identified for significance.

## Results

### Serum HBsAg and HBeAg expression in PPAR agonists treated HBV replicative mice

All serum HBsAg were identified positive. The average serum HBsAg level in saline, bezafibrate, fenofibrate and rosiglitazone groups were (2.387 ± 0.078) log_10_ IU/mL, (2.870 ± 0.070) log_10_ IU/mL, (2.860 ± 0.015) log_10_ IU/mL and (3.040 ± 0.042) log_10_ IU/mL respectively. It showed that the serum HBsAg was significantly increased after PPAR agonists treatment (*p* < 0.05).

**Fig. 1:**
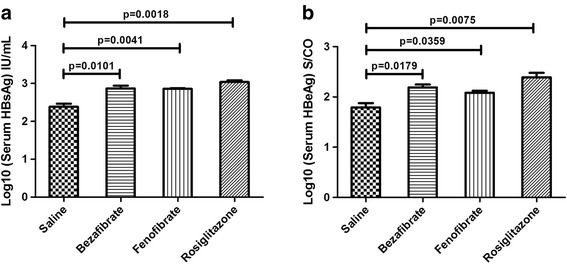
The alternation of serum HBV antigens in mouse models after PPAR agonists treatment. HBsAg and HBeAg levels in the mouse sera were measured through ECLIA. The mean HBsAg and HBeAg levels plus standard deviation (indicated by *error bars*) were shown. Panel **a** showed the alternation of serum HBsAg. Panel **b** showed the alternation of serum HBeAg

Similarly, positive HBeAg were found in all samples. The average serum HBeAg level were (1.792 ± 0.084) log_10_ S/CO in saline group, (2.189 ± 0.058) log_10_ S/CO in bezafibrate group, (2.084 ± 0.041) log_10_ S/CO in fenofibrate group and (2.392 ± 0.085) log10 S/CO in rosiglitazone group. Obviously, the serum HBeAg was also significantly increased by the three PPAR agonists (*p* < 0.05) (Fig. 1).

### Serum HBV DNA in PPAR agonists treated HBV replicative mice

**Fig. 2:**
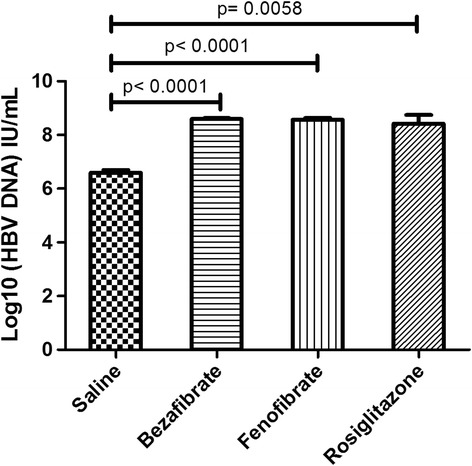
The alternation of serum HBV DNA in mouse models after PPAR agonists treatment. Serum HBV DNA load in the mouse sera were measured through real time PCR (Fluorescence Probing). The mean serum HBV DNA load plus standard deviation (indicated by *error bars*) were shown

Four standard samples in the diagnostic kit with fixed viral load were amplified to establish a standard curve (y = -0.2943x + 13.194 (IU/mL), R^2^ = 0.998). With the standard curve, viral loads in mouse serum samples were calculated and analyzed. In three groups treated with PPAR agonists. The average viral load in bezafibrate, fenofibrate and rosiglitazone groups were (8.594 ± 0.037) log_10_ IU/mL, (8.571 ± 0.063) log_10_ IU/mL and (8.420 ± 0.326) log_10_ IU/mL respectively, while the average viral load in saline group was (6.595 ± 0.098) log_10_ IU/mL. Serum viral load was significantly elevated after PPAR agonist treatment (*p* <0.05) (Fig. 2).

### HBV replication intermediates in liver tissues of PPAR agonists treated HBV replicative mice

**Fig. 3:**
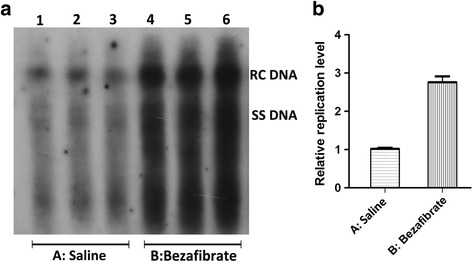
The alternation of HBV replication intermediates in mouse models after bezafibrate treatment. Mice were injected hydrodynamically with 10 μg pHBV4.1 and treated with bezafibrate for 3 days. HBV replication intermediates were detected through DNA (Southern) filter hybridization. Panel **a** is the representative image of detected HBV replication intermediates, and Panel **b** is its statistical diagram after the qualification of all bands in three independent experiments

**Fig. 4:**
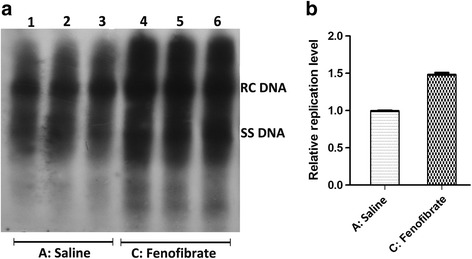
The alternation of HBV replication intermediates in mouse models after fenofibrate treatment. Mice were injected hydrodynamically with 10 μg pHBV4.1 and treated with fenofibrate for 3 days. HBV replication intermediates were detected through DNA (Southern) filter hybridization. Panel **a** is the representative image of detected HBV replication intermediates, and Panel **b** is its statistical diagram after qualification of all bands in three independent experiments

**Fig. 5:**
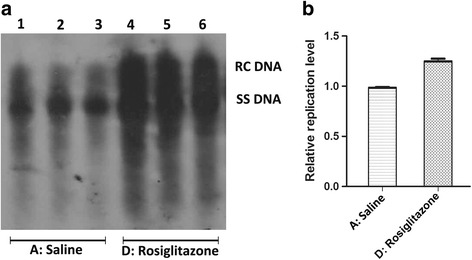
The alternation of HBV replication intermediates in mouse models after rosiglitazone treatment. Mice were injected hydrodynamically with 10 μg pHBV4.1 and treated with rosiglitazone for 3 days. HBV replication intermediates were detected through DNA (Southern) filter hybridization. Panel **a** is the representative image of detected HBV replication intermediates, and Panel **b** is its statistical diagram after qualification of all bands in three independent experiments

HBV replication intermediates analysis was performed in all groups. The results of three independent experiments showed that the level of HBV replication intermediates were increased significantly by the three PPARs agonists. Quantitative analysis of the detected replication intermediates showed that bezafibrate could increase the viral replication intermediates up to about 2.7 folds (Fig. 3). Fenofibrate presented a relatively moderate effect, it could increase the viral replication intermediates up to about 1.5 folds (Fig. 4). Rosiglitazone also increased the viral replication in a moderate way and it could increase HBV replication intermediates up to about 1.3 folds (Fig. 5). These results suggested a risk in the activation of HBV replication when PPAR agonists were used for HBV infected metabolic disease patients.

### HBV transcription in liver tissues of PPAR agonists treated HBV replicative mice

**Fig. 6:**
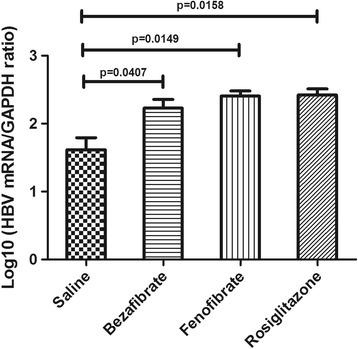
The alternation of HBV transcription level in mouse models after PPAR agonist treatment. The ratio of HBV mRNA versus GAPDH mRNA was applied to indicated HBV transcription level. And logarithm was taken. The Log_10_(HBV mRNA/GAPDH ratio) in each group was shown

The ratio of HBV mRNA versus GAPDH mRNA was applied to indicate HBV transcription level in mouse livers. After logarithm was taken, it turned out the Log_10_ (HBV mRNA/GAPDH ratio) in saline groups was 1.62 ± 0.18, while in bezafibrate group, fenofibrate group and rosiglitazone group, they were 2.23 ± 0.13, 2.40 ± 0.07,2.42 ± 0.09 respectively. The HBV mRNA levels in the bezafibrate group, fenofibrate group and rosiglitazone group were significantly higher than that in the saline group (*p* < 0.05). It implied that these three PPAR agonists could activate HBV replication at transcription level (Fig. 6).

### Hepatic HBcAg expression in PPAR agonists treated HBV replicative mice

**Fig. 7:**
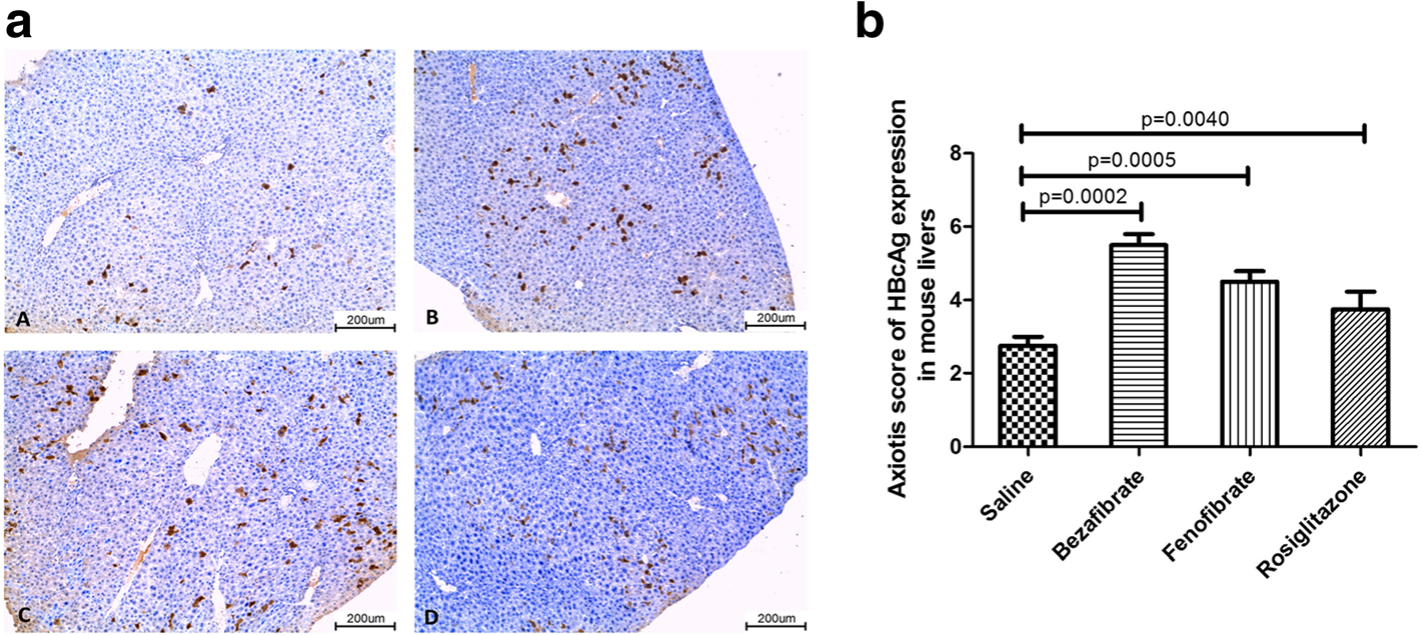
Immunohistochemical staining of HBcAg in the livers of HBV replicative models. Positive stain of HBcAg wa﻿s observed in the livers. Representative images in four groups were shown in Panel **a** *A-A*: Saline; *A-B*: Bezafibrate; *A-C*: Fenofibrate; and *A-D*: Rosiglitazone. And the statistical diagram of Axiotis Score of HBcAg expression in each group was shown in the Panel **b**

Positive HBcAg expression was identified in all HBV replicative mouse models. The score of HBcAg expression in mouse livers significantly increased when PPAR agonists were administrated, no matter in bezafibrate, fenofibrate or rosiglitazone group (Fig. 7). It suggested that PPAR agonists administrated at the dose equivalent to clinical usage could activate expression of HBcAg in mouse livers, after up-regulation of HBV replication.

## Discussion

Patients with metabolic disorders could benefit from the administration of the synthetic agonists of PPARs [16, 17]. For example, fibrates has been proved to decrease risks of developing cardiovascular diseases from atherogenic dyslipidemia [18]. And thiazolidinediones showed promising effect in reducing major adverse cardiovascular events in patients with T2DM [19]. However, as one of the liver-enriched transcriptional factors, PPARs is related to HBV replication. PPARα worked as a ligand to support viral biosynthesis when clofibric acid activated HBV transcription and replication [20]. And PPARγ coactviator 1α (PGC1α) enhanced viral biosynthesis via its nuclear receptors [21]. Several studies have confirmed that the activation of PPAR potentiated viral replication in vitro and in vivo [8–10]. So the situation becomes complicated when patients receiving PPAR agonists have HBV infection.

The possible side effect of PPAR agonists to HBV replication could not be neglected according to the previous studies. But these studies were mainly implemented with experimental agents other than clinically used drug. Moreover, each study focused on one isolate of PPARs only. A systematic study on different kinds of PPARs needs to be supplemented.

In our study, we chose three representative synthetic agonists activating different isolates of PPARs including bezafibrate of pan PPARs agonists, fenofibrate of PPARα agonists and rosiglitazone of PPARγ agonists. And we applied a previously used hydrodynamic HBV replicative mouse model to learn their influence to viral replication. Obviously, viral transcription, replication, and antigen expression were increased by these drugs. And pan-agonists would increase viral replication and HBcAg expression more effectively. Thus a systematic profile on the drug-induced effect to viral replication were acquired in vivo.

Fenofibrate belongs to the PPARα agonists. Another PPARα agonist, clofibrate, has been confirmed to increase viral replication both in HBV transiently transfected Huh7 cells and transgenic HBV mouse models [8, 9]. And miR-141, a specific interfering micro RNA targeting on PPARα, would inhibit HBV replication effectively [22]. Now PPARα is studied as a potential antiviral target. Along with these studies, our findings have clarified its role as an essential promoting factor in viral replication and expression.

Rosiglitazone is a classic PPARγ agonist. Studies in vitro found 15-deoxyprostaglandin J2 (15d-PGJ2) could activate PPARγ to increase viral replication in HBV transiently transfected cell modes. Theoretically, rosiglitazone should have the same effect. But the actual findings remained controversial. Some researchers found that it activated viral replication in kinds of HBV transfected hepatocellular carcinoma cells while the others found that it would inhibit viral replication in vitro [10, 11]. To settle the arguments, rosiglitazone was studied in HBV replicative mouse model, and its activating effect was found. Besides direct activation via PPARγ, rosiglitazone was also considered as a enhancer of HBV DNA polymerase by some researchers [10]. It could be another way to increase HBV replication.

As a pan-agonist, bezafibrate activates PPARα, PPAR β/δ, as well as PPARγ. Naturally, it was supposed to increase HBV replication. But Wakui’s group brought out a different opinion [11]. According to their study, bezafibrate had no effect on viral replication in HBV replicative cell lines. In their opinion, PPARα played an activating role while PPARγ played an inhibitory role. And PPARα and PPARγ agnoists worked reciprocally. On the contrary, we found that bezafibrate would activate HBV replication in vivo. And it showed stronger effect than fenofibrate or rosiglitazone as pan-agonists could activate multiple PPARs to result in a stronger activation on HBV replication.

Different from previous studies which applied laboratory biochemical agents as PPAR agonists [8–10], the experimental drugs here were marketed drugs used in clinical practice. And the dosage of drugs applied in experiments was equivalent to clinical dosage. Under such conditions, our study mimicked clinical situation in mouse models. The results gives a much persuasive evidence that clinically these drugs would work as PPAR agonists to activate viral replication. And if a patient has HBV infection, even when PPAR agonists were used in regular dosage for treating metabolic disorder, risk of hepatitis exacerbation may occur. W﻿e also tried dose-dependent effect of the three agonists in vivo. And we found that when we doubled the dosage of the agonists administrated in HBV replicative mouse models, the HBV replication levels also showed significant elevation through Southern Blot (Additional file 1: Supplementary Figure 1).

The World Health Organization (WHO) forecasts that there would be more than 300 million patients suffering diabetes and related metabolic diseases. And over 90% cases would be type 2 diabetes which is featured by insulin deficiency and insulin resistance. PPARs agonists show promising effect in improving metabolic disorders including obesity, hypertension, diabetes and cardiovascular diseases and are widely used in practice. Moreover, agonists are developing rapidly from partial agonists to dual agonists, even pan-agonists [23, 24]. However, the more effective the drugs were, the more possibilities they would brought to the activation of viral replication in HBV infected patients. Our findings provide useful information for well management in these patients.

## Conclusion

Our findings solved controversies on PPARs’ role in HBV viral replication and supplemented more data on PPARs agonists induced viral replication. By activating different PPARs in vivo, we found that synthetic PPARs pan-agonist bezafibrate, PPARα agonist fenofibrate and PPARγ agonist rosiglitazone would increase HBV transcription, replication and antigens. Meanwhile, our findings suggested that when HBV infected patients were treated with PPARs agonists because of metabolic diseases, HBV viral load should be monitored. The regimens may need to be adjusted or an antiviral therapy maybe added if necessary.

## Additional file

Additional file 1: Supplementary Figure 1. Alternation of HBV replicative intermediates in mouse livers after three PPAR agonists treatment. A. Alternation of HBV replicative intermediates in mouse livers after bezafibrate treatment at 30mg/kg.d and 60 mg/kg.d. B. Alternation of HBV replicative intermediates in mouse livers after fenofibrate treatment at 30mg/kg.d and 60 mg/kg.d. C. Alternation of HBV replicative intermediates in mouse livers after rosiglitazone treatment at 0.6mg/kg.d and 1.2 mg/kg.d. (DOCX 169 kb).

## Abbreviations

15d-PGJ2: 15-deoxyprostaglandin J2
DAB: 3' 3'-diaminobenzidine tetrahydrochloride
ECLIA: Electrochemiluminescence immunoassay
HBV: Hepatitis B virus
IHC: Immunohistochemistry
PGC1α: PPARγ coactviator 1α
PPAR: Peroxisome proliferators activated receptor
T2DM: Type 2 diabetes mellitus
