# Supplementary material for: Peroxisome Proliferators Activated Receptor (PPAR) agonists activate hepatitis B virus replication in vivo
Source: Virol J. 2017 May 25;14:96. doi: 10.1186/s12985-017-0765-x (PMC5445479; doi:10.1186/s12985-017-0765-x)
Supplement: Additional file 1: — Supplementary Figure 1. Alternation of HBV replicative intermediates in mouse livers after three PPAR agonists treatment. A. Alternation of HBV replicative intermediates in mouse livers after bezafibrate treatment at 30mg/kg.d and 60 mg/kg.d. B. Alternation of HBV replicative intermediates in mouse livers after fenofibrate treatment at 30mg/kg.d and 60 mg/kg.d. C. Alternation of HBV replicative intermediates in mouse livers after rosiglitazone treatment at 0.6mg/kg.d and 1.2 mg/kg.d. (DOCX 169 kb). [file 12985_2017_765_MOESM1_ESM.docx]

We have tried dose-dependent effect of the three agonists in vivo. And we found that when we doubled the dosage of the agonists administrated in HBV replicative mouse models, the HBV replication levels also showed significant elevation through Southern Blot (Figure 1). If both the editor and reviewer suggest it is necessary, we can present the result in a supplementary file.


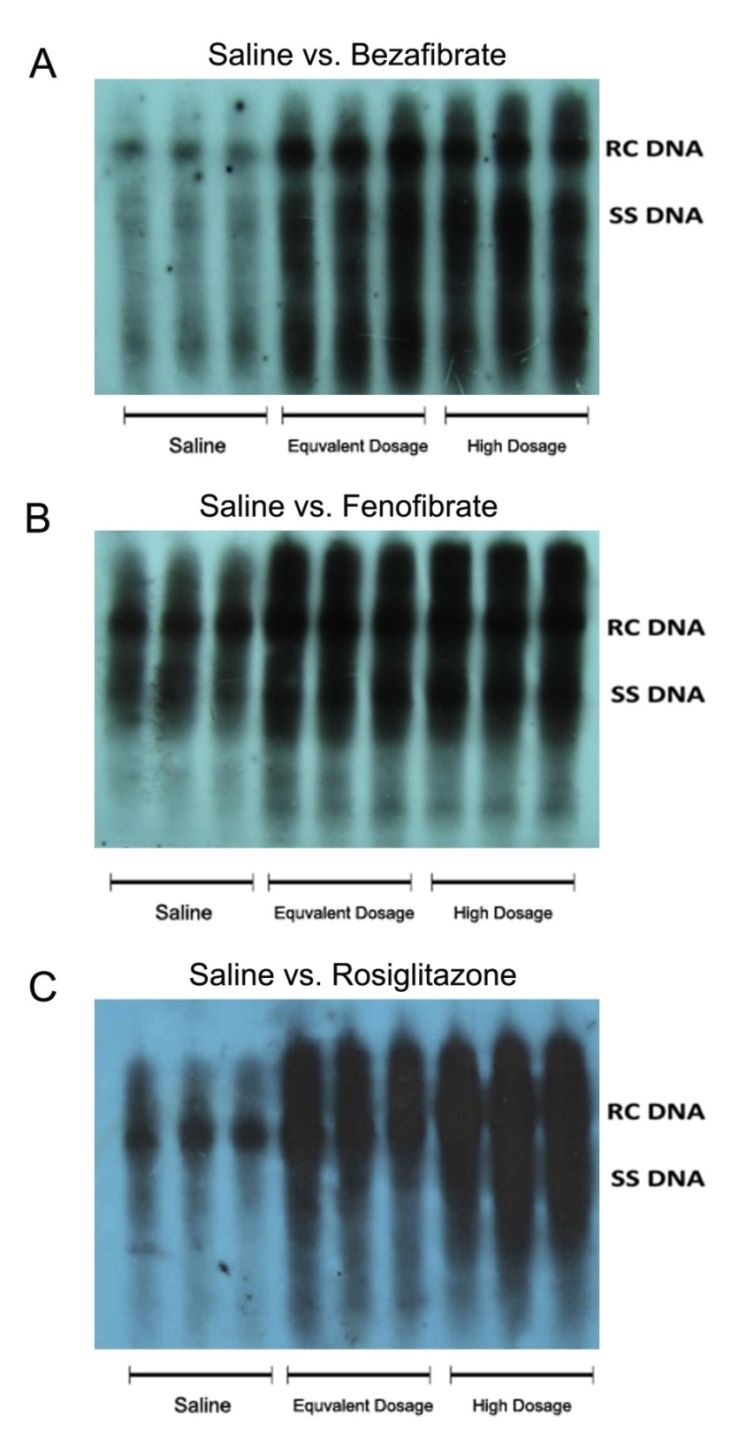


Figure S1. Alternation of HBV replicative intermediates in mouse livers after three PPAR agonists treatment.

A. Alternation of HBV replicative intermediates in mouse livers after bezafibrate treatment at 30mg/kg.d and 60 mg/kg.d.

B. Alternation of HBV replicative intermediates in mouse livers after fenofibrate treatment at 30mg/kg.d and 60 mg/kg.d.

C. Alternation of HBV replicative intermediates in mouse livers after rosiglitazone treatment at 0.6mg/kg.d and 1.2 mg/kg.d.
